# External Ventricular Drainage for Hydrocephalus Following Cerebellar Infarction: A Scoping Review

**DOI:** 10.3390/jcm14248663

**Published:** 2025-12-06

**Authors:** Tatsuya Tanaka, Eiichi Suehiro, Akira Matsuno

**Affiliations:** Department of Neurosurgery, International University of Health and Welfare Narita Hospital, 852 Hatakeda, Narita 2868520, Japan; esuehiro@iuhw.ac.jp (E.S.);

**Keywords:** cerebellar infarction, obstructive hydrocephalus, external ventricular drainage, suboccipital decompression, upward herniation, scoping review

## Abstract

**Background**: Cerebellar infarction complicated by obstructive hydrocephalus is a life-threatening condition. External ventricular drainage (EVD) has traditionally been regarded as hazardous due to concerns about precipitating upward transtentorial herniation, whereas suboccipital decompressive craniectomy (SDC) remains the definitive life-saving treatment. The optimal role and sequencing of these interventions remain controversial. **Methods**: A scoping review was conducted in accordance with PRISMA-ScR guidelines. PubMed/MEDLINE was systematically searched from inception to September 2025. Eligible studies included adult patients with cerebellar infarction and acute obstructive hydrocephalus managed with EVD, with or without SDC. Data on study design, patient characteristics, interventions, complications, and outcomes were extracted and narratively synthesized. **Results**: Forty studies were included, encompassing multicenter registries, retrospective cohorts, case series, and international guidelines. Evidence suggests that EVD alone can be effective in selected patients with preserved or moderately impaired consciousness, while outcomes in comatose patients are improved with SDC or combined approaches. Importantly, this scoping review integrates current evidence with a representative institutional case to provide a practical clinical context. Radiographic signs of upward transtentorial herniation before EVD were common, but clinically significant deterioration was infrequent. Prognostic factors for surgical decision-making included infarct volume (practical threshold 25–35 mL), location (vermian or bilateral infarcts), brainstem involvement, and level of consciousness. International guidelines increasingly recognize EVD as a valid treatment option, particularly as initial therapy for hydrocephalus. **Conclusions**: EVD should no longer be regarded as an absolute contraindication in cerebellar infarction with obstructive hydrocephalus. Controlled drainage can suffice in carefully selected patients, whereas SDC remains indispensable in cases with severe mass effect or brainstem compression. A pragmatic stepwise approach—beginning with cautious EVD and escalating to SDC when indicated—may optimize outcomes. Further multicenter studies are required to refine patient selection criteria and establish standardized management algorithms.

## 1. Introduction

Cerebellar infarction accounts for approximately 1.5–10% of all ischemic strokes and is associated with high morbidity and mortality, particularly when complicated by obstructive hydrocephalus or brainstem compression [1,2,3]. Because the posterior fossa has a limited anatomical volume, patients are prone to rapid neurological deterioration, and prompt surgical intervention is crucial to prevent fatal herniation syndromes.

Two main surgical strategies have been described: external ventricular drainage (EVD), which provides rapid cerebrospinal fluid (CSF) diversion, and suboccipital decompressive craniectomy (SDC), which directly relieves cerebellar swelling and obstructive hydrocephalus. Although EVD offers immediate CSF decompression, it has historically been regarded with caution due to concerns about precipitating upward transtentorial herniation, leading to reluctance in its use as a sole therapy [3,4,5]. In contrast, SDC is more invasive and may not always be immediately feasible depending on institutional resources and patient comorbidities.

International guidelines have also evolved over time. The 2014 AHA/ASA scientific statement recommended that EVD for obstructive hydrocephalus after cerebellar infarction generally be accompanied by or followed with SDC [3]. Subsequently, the AHA/ASA guideline update (2019) specified that EVD is recommended for obstructive hydrocephalus (Class I, C-LD), while SDC should be performed in patients with neurological deterioration from brainstem compression despite maximal medical therapy (Class I, B-NR) [6]. More recently, the ESO 2021 guideline stated that drainage of CSF (e.g., EVD), alone or in combination with SDC, may be considered in selected patients, while emphasizing ongoing uncertainty regarding patient selection and timing [7].

Observational studies have further informed clinical practice. Raco et al. reported favorable outcomes in carefully selected patients managed with EVD alone, suggesting that immediate decompression is not invariably required [8]. Conversely, other reports emphasized that early SDC improves survival in comatose patients with severe mass effect [9,10]. Importantly, a recent retrospective analysis demonstrated that while radiographic signs of upward herniation were present in 88% of patients prior to EVD, only 8% deteriorated clinically after drainage [11]. These findings challenge the traditional assumption that EVD is inherently dangerous and suggest that the risks may have been overstated.

Taken together, the role of EVD in cerebellar infarction complicated by obstructive hydrocephalus remains a matter of debate. A systematic synthesis of existing evidence is required to clarify its indications, safety, and clinical role. The present scoping review aims to (1) comprehensively map the evidence on the use of EVD in acute obstructive hydrocephalus secondary to cerebellar infarction, (2) contextualize its role relative to SDC, and (3) reinforce its clinical relevance by presenting a representative case from our institution. We have clarified that the focus of this study is specifically on EVD in cerebellar infarction complicated by obstructive hydrocephalus, rather than on all interventional treatments for space-occupying posterior fossa infarction. This narrower scope reflects our intention to address the long-standing perception that EVD is inherently contraindicated in posterior fossa pathology, irrespective of hydrocephalus mechanism or progression pattern. By doing so, we aim to provide insights that may inform clinical decision-making in this challenging condition.

## 2. Methods

This scoping review was conducted and reported in accordance with the PRISMA Extension for Scoping Reviews (PRISMA-ScR) [12]. No review protocol was registered for this study. A comprehensive search of the PubMed database was performed from its inception to 25 September 2025, using the following strategy: (“cerebellar infarction” [Title/Abstract] OR “cerebellar stroke” [Title/Abstract]) AND (hydrocephalus OR “fourth ventricle” OR obstructive) AND (“external ventricular drainage” OR ventriculostomy OR EVD OR “CSF drainage” OR “cerebrospinal fluid”). Additional keywords included suboccipital decompressive craniectomy, decompression, upward herniation, transtentorial, endoscopic third ventriculostomy (ETV), and shunt. Citation tracking was also performed using major international guidelines, including the AHA/ASA scientific statement on cerebral and cerebellar infarction with swelling [3], the 2018 AHA/ASA guideline and its 2019 update on acute ischemic stroke management [6,13], and the ESO guideline on space-occupying brain infarction [7].

Eligible studies included adult patients with cerebellar infarction (including those with hemorrhagic transformation) complicated by acute obstructive hydrocephalus, fourth ventricle narrowing or obliteration, or brainstem compression. Interventions of interest were external ventricular drainage (EVD), either alone or in combination with suboccipital decompressive craniectomy (SDC), endoscopic third ventriculostomy (ETV), or other surgical procedures. Outcomes included mortality, functional status assessed using the modified Rankin Scale (mRS), Glasgow Outcome Scale (GOS) or its extended version (GOS-E), upward transtentorial herniation, infection, reoperation, and the need for long-term cerebrospinal fluid (CSF) diversion. Retrospective cohort studies, case series, case reports, clinical guidelines, and narrative or systematic reviews were included, while studies focusing solely on cerebellar hemorrhage, pediatric populations, animal experiments, and conference abstracts without full text were excluded.

**Figure 1:**
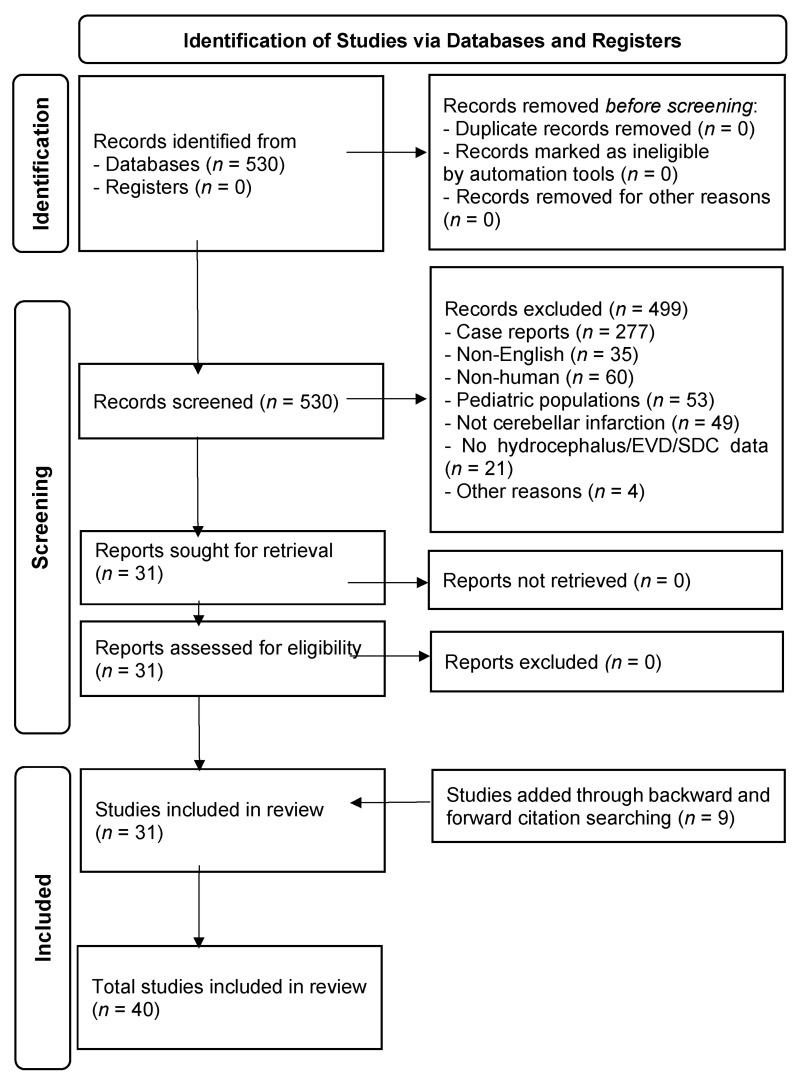
PRISMA flow diagram for scoping review.

Screening and data extraction were performed independently by the author (T.T.). Titles and abstracts were initially reviewed for eligibility, followed by full-text assessment when necessary. Data were charted using a standardized Excel spreadsheet and included study characteristics (first author, year, design, sample size), patient demographics, radiological findings (fourth ventricle narrowing, brainstem compression, upward herniation), interventions (EVD, concomitant procedures, surgical timing), complications (infection, upward herniation, reoperation), and outcomes (mortality, functional recovery at discharge or follow-up). Given the considerable heterogeneity among studies, quantitative synthesis was not performed; instead, a narrative mapping approach was adopted. The screening process is summarized in the PRISMA-ScR flow diagram (Figure 1). In total, 40 studies met the inclusion criteria and were analyzed in this review.

In addition to literature synthesis, one representative case from our institution is illustrated.

## 3. Results

### 3.1. Study Selection and Characteristics

The PubMed search identified a total of 530 records. After screening, case reports (*n* = 277), non-English articles (*n* = 35), non-human studies (*n* = 60), pediatric studies (*n* = 53), studies not involving cerebellar infarction (*n* = 49), studies without data on hydrocephalus or EVD/SDC (*n* = 21), and other irrelevant reports (*n* = 4) were excluded. Therefore, 31 articles underwent full-text assessment, and an additional 9 were identified through citation tracking, resulting in a total of 40 studies included in this review (Figure 1). Single case reports were excluded from the quantitative synthesis but are narratively discussed in the Section 4.

The included studies ranged from the late 1970s to the 2020s and were conducted primarily in Europe, North America, and Asia. Study designs comprised multicenter registries [1,4,14,15,16,17,18,19,20,21,22,23,24], single-center retrospective cohorts or case series [8,9,10,11,25,26,27,28,29,30,31,32,33,34,35,36,37,38,39,40,41,42,43], and focused analyses on the safety of EVD [11]. No randomized controlled trials were identified, and the overall quality of evidence was low. Several review articles were also included [2,5,25,44,45], as well as international guideline statements [3,6,7,13].

A summary of the included studies, including patient demographics, imaging features, interventions, outcomes, and observations of upward herniation, is provided in Supplementary Table S1. The following Section 3.2, Section 3.3, Section 3.4, Section 3.5 synthesize the clinical insights and interpretative findings derived from these data.

### 3.2. Evaluation of EVD and Comparative Outcomes

#### 3.2.1. Potential Efficacy of EVD Alone

Several studies demonstrated that EVD alone may provide adequate management in selected patients. Raco et al. reported favorable outcomes in seven of eight patients treated with EVD without decompression [8]. Similarly, Lim et al. observed that stable cases could be managed conservatively or with EVD, whereas severe cases required decompressive surgery [44].

#### 3.2.2. Superiority of SDC in Severe Cases

In comatose patients with brainstem compression, SDC showed superior outcomes compared with EVD alone [9]. The German–Austrian registry identified the level of consciousness at presentation as the most critical prognostic factor, with surgical interventions (SDC ± EVD) improving survival and functional outcomes in severe cases [1,14]. Mostofi et al. also demonstrated significantly better survival and functional recovery among patients undergoing surgical treatment (EVD/SDC) compared with conservative therapy [10].

#### 3.2.3. Safety and Risk of Upward Herniation

Braksick et al. found radiographic evidence of upward herniation in 22 of 25 patients (88%) prior to EVD placement, yet clinical deterioration after drainage occurred in only 8%, indicating that EVD is not contraindicated when drainage is carefully titrated (e.g., higher threshold settings, limited outflow) [11].

#### 3.2.4. Alternative or Adjunctive Measures

Endoscopic third ventriculostomy (ETV) was effective in selected patterns of obstruction [34], although evidence remains limited. Several studies also noted the need for long-term CSF diversion (e.g., ventriculoperitoneal shunt) during the chronic phase.

### 3.3. Surgical Indication Factors

#### 3.3.1. Infarct Volume

A multicenter matched analysis showed that infarct volumes ≥ 35 mL were associated with improved outcomes after surgical decompression, while infarcts < 25 mL were better managed conservatively [22]. Hernandez-Duran et al. proposed a ROC-derived cutoff of approximately 31.3 cm^3^ for SDC consideration [23]. Taylor et al. developed a deterioration risk score incorporating infarct volume, swelling ratio, and posterior fossa volume [44].

#### 3.3.2. Location

Vermian infarctions leading to fourth ventricle compression and bilateral cerebellar involvement were consistently identified as high-risk features [1,39].

Brainstem involvement

The presence of concurrent brainstem infarction strongly predicted poor outcomes [39].

#### 3.3.3. Clinical Presentation

Depressed consciousness and brainstem signs—such as pupillary abnormalities, oculomotor deficits, and respiratory disturbances—were consistent triggers for surgical intervention [1,9,10].

Summary

Overall, surgical candidacy should be determined not solely by the presence of hydrocephalus but through a multidimensional assessment incorporating infarct volume (typically ≥ 25–35 mL), anatomical location, brainstem involvement, and neurological status.

### 3.4. Guideline Perspective

#### 3.4.1. AHA/ASA 2014

Recommended EVD for obstructive hydrocephalus, with prompt SDC in cases of mass effect or neurological deterioration [3].

#### 3.4.2. AHA/ASA 2018/2019

Upgraded EVD to a Class I, C–LD recommendation, and advised SDC for patients with brainstem compression or worsening neurological status [6,13].

#### 3.4.3. ESO 2021

Permitted the use of EVD alone or in combination with SDC, while emphasizing the very low level of evidence supporting either approach [7].

### 3.5. Representative Case Description

An 80-year-old man with a history of atrial fibrillation and hypertension, who was independent in activities of daily living (modified Rankin Scale [mRS] score, 0), presented to a local hospital with acute onset of dizziness and gait instability on day 0. On hospital day 1, his symptoms worsened, accompanied by nausea and vomiting. Brain imaging revealed a right cerebellar infarction with hemorrhagic transformation and acute hydrocephalus, prompting transfer to our hospital for further management. His medications included rivaroxaban, aspirin, dipyridamole, and candesartan. He was a nonsmoker and did not consume alcohol.

**Figure 2:**
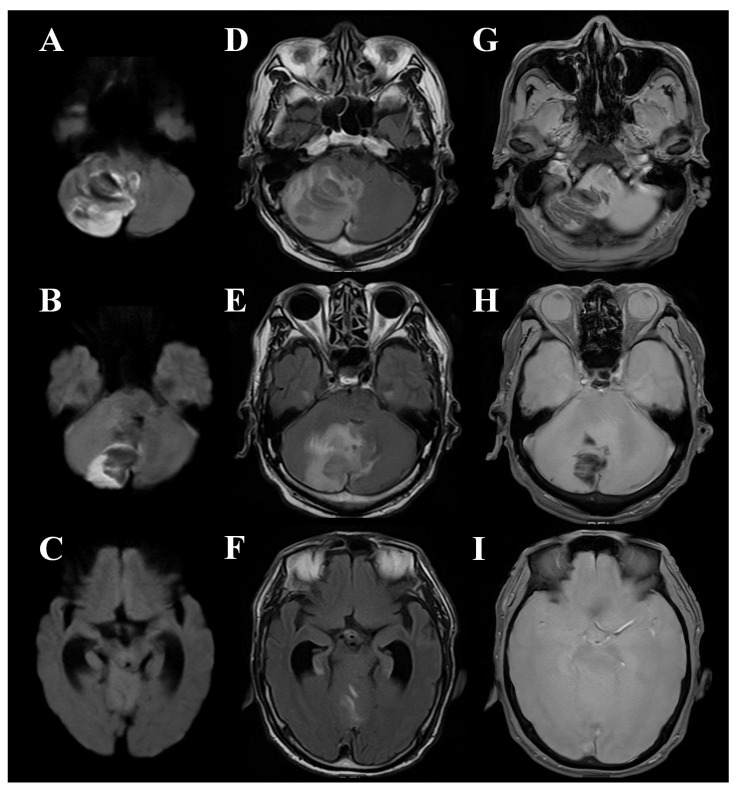
Magnetic resonance imaging findings on admission. (**A**–**C**) Diffusion-weighted imaging (DWI) demonstrates an acute right cerebellar infarction with a high signal intensity lesion in the posterior inferior cerebellar hemisphere, associated with mass effect compressing the fourth ventricle. (**D**–**F**) Fluid-attenuated inversion recovery (FLAIR) images show hyperintense signals in the infarcted area with surrounding edema and mild hemorrhagic transformation. (**G**–**I**) T2*-weighted images reveal patchy low-signal areas within the infarcted region, indicating hemorrhagic components.

**Figure 3:**
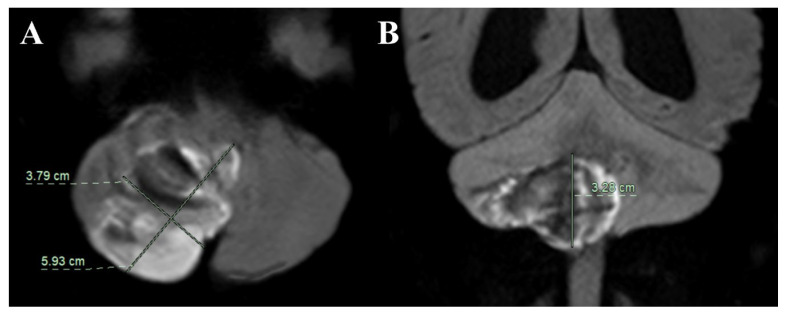
Measurement of infarct volume on diffusion-weighted imaging. (**A**) Axial DWI showing the maximum lesion diameter (5.93 × 3.79 cm) in the right cerebellar hemisphere. (**B**) Coronal DWI image demonstrating the craniocaudal extent (3.28 cm). The infarct volume was estimated to be approximately 38.8 mL using a simplified ellipsoid formula.

On admission (day 1), the Glasgow Coma Scale (GCS) score was E3V4M6. Vital signs were as follows: blood pressure, 179/90 mmHg; heart rate, 105 beats/min; body temperature, 37.6 °C; and oxygen saturation, 99% on 2 L/min oxygen via nasal cannula. Neurological examination revealed no apparent limb weakness. Computed tomography (CT) and magnetic resonance imaging (MRI) demonstrated a right cerebellar infarction with hemorrhagic changes, fourth ventricular compression, obliteration of the quadrigeminal cistern, and dilatation of the lateral ventricles, consistent with acute obstructive hydrocephalus. No brainstem infarction was identified (Figure 2A–C). The estimated infarct volume, calculated using a simplified method, was approximately 38.8 mL (Figure 3). Magnetic resonance angiography showed no evidence of large-vessel occlusion. As the patient had taken rivaroxaban the previous day, no reversal agent was administered.

**Figure 4:**
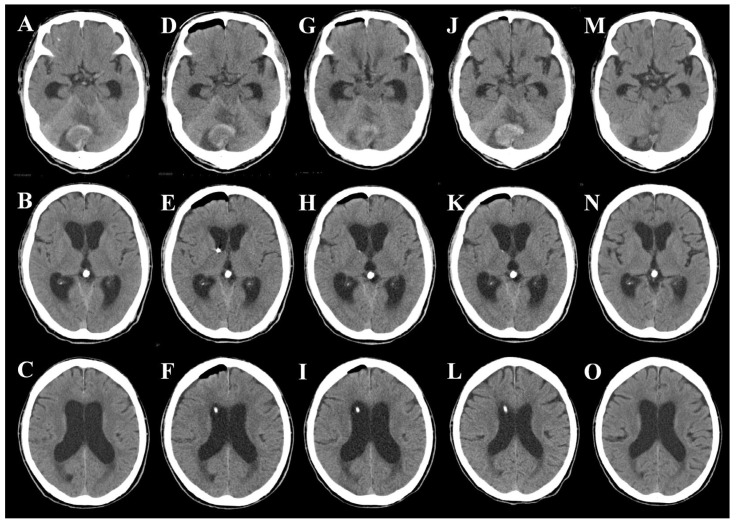
Serial computed tomography (CT) scans during hospitalization. (**A**–**C**) Day 1 (before EVD): CT showing right cerebellar infarction with fourth ventricular compression and acute obstructive hydrocephalus. (**D**–**F**) Day 1 (after EVD): CT confirming appropriate ventricular catheter placement with partial reduction in ventricular size. (**G**–**I**) Day 2: Persistent ventricular dilatation due to limited drainage, later improved following additional cerebrospinal fluid removal. (**J**–**L**) Day 6: CT after EVD removal showing maintained ventricular size without new hemorrhage. (**M**–**O**) Day 26: Follow-up CT demonstrating resolved hydrocephalus and reduction in mass effect in the posterior fossa.

Medical management, including antihypertensive therapy, hemostatic agents, and anti-edema treatment, was initiated. However, his level of consciousness gradually declined, with the GCS decreasing to E1V1M4. The impaired consciousness was presumed to result from progressive hydrocephalus or direct brainstem compression. To clarify the predominant mechanism and alleviate intracranial pressure, emergency EVD was performed via the right frontal horn under local anesthesia on day 1 (Figure 4D–F). The drainage system was set at +15 cm H_2_O above the external auditory canal, allowing intermittent CSF drainage. Following the procedure, the GCS improved rapidly to E3V4M6.

On day 2, transient deterioration (E3V2M6) was observed, and CT demonstrated persistent ventricular dilatation; however, the neurological status promptly improved after additional CSF drainage (Figure 4G–I). To avoid excessive pressure gradients, the EVD was maintained at a high threshold and removed on day 6 (Figure 4J–L). Anticoagulation with edoxaban was resumed on day 7. During hospitalization, the patient developed aspiration pneumonia, which responded well to antibiotic therapy. Notably, no clinical signs of upward transtentorial herniation were observed, and decompressive craniectomy was not required.

## 4. Discussion

Surgical management of obstructive hydrocephalus secondary to cerebellar infarction remains a subject of ongoing debate. Historically, EVD for posterior fossa lesions such as cerebellar tumors or hemorrhage was regarded as a hazardous procedure because of the presumed risk of precipitating upward transtentorial herniation. This view was primarily based on early reports [46,47]. By extension, the same concern was applied to cerebellar infarction, with several authors recommending that EVD should only be performed in conjunction with, or immediately prior to, suboccipital decompression [31]. However, these early reports made it difficult to determine whether clinical deterioration was truly caused by abrupt CSF pressure gradients after drainage, or rather by progressive cerebellar swelling and direct brainstem compression. Consequently, the long-standing notion that “EVD is inherently dangerous” may have been overstated.

From a neuroradiological perspective, the definition of upward transtentorial herniation remains inconsistent. Although it was first described on CT in 1978 [48], no universally accepted diagnostic criteria have since been established. Typical radiological markers include obliteration of the quadrigeminal cistern, effacement of the fourth ventricle, and midbrain deformation [1,14]. Clinically, however, it is often extremely difficult to differentiate symptoms due to intracranial hypertension from hydrocephalus versus direct brainstem compression [16,27].

Early studies in the literature described several cases of neurological deterioration or presumed upward herniation following EVD in patients with extensive infarcts and brainstem compression, underscoring the fact that drainage is not universally safe. More recent reports have shown that, with careful patient selection and controlled drainage, EVD can stabilize or improve outcomes in selected cases. A staged approach—initial control of hydrocephalus with EVD, followed by SDC if no improvement is observed—has been advocated [8]. In contrast, SDC was shown to be superior to EVD alone in comatose patients [9], and the German–Austrian registry emphasized that the strongest predictor of outcome was the level of consciousness at presentation, with severely affected patients benefitting most from decompressive surgery [1,14]. Similarly, significantly better outcomes were reported in surgically treated patients (EVD or SDC) compared with those managed conservatively [10].

Regarding safety, radiographic signs of upward herniation were frequently present prior to EVD placement; however, only a small proportion of patients showed clinical deterioration after drainage [11]. This suggests that EVD is not inherently contraindicated and can be performed safely when drainage is cautiously titrated—initiating at a higher threshold and avoiding excessive outflow. Consistently, international guidelines have recommended EVD for obstructive hydrocephalus as a Class I, C-LD intervention [6,13]. Similarly, the ESO guideline indicated that EVD alone or in combination with SDC may be considered in selected patients, though it emphasized that the overall quality of evidence remains very low [7]. In contemporary practice, many centers aim to limit abrupt pressure shifts by maintaining the EVD system at approximately +5–15 cm H_2_O above the external auditory canal and avoiding rapid cerebrospinal fluid outflow (typically <5–20 mL per hour, with sustained drainage above ~20 mL/h considered overdrainage and a trigger to raise the set level). These parameters represent common clinical practice rather than standardized guidelines and are intended to minimize large supratentorial–infratentorial pressure gradients and the theoretical risk of upward transtentorial herniation.

SDC, in contrast, is widely recognized as life-saving, though its evidence base derives mainly from retrospective observational studies. Favorable outcomes have been observed in relatively young patients without brainstem involvement, while elderly patients and those with concurrent brainstem infarction frequently experience poor prognoses [19,39]. Other studies, while suggesting that SDC may reduce mortality, have not consistently demonstrated improvements in good functional outcomes such as mRS 0–2.

Cerebellar infarct volume has emerged as a critical determinant in surgical decision-making. Infarcts larger than 35 cm^3^ have been associated with poor prognosis, whereas postoperative volumes ≤ 17 cm^3^ or reductions ≥ 50% correlate with favorable outcomes [23,25]. Volumes exceeding 25–30 cm^3^ have similarly been linked to unfavorable prognosis [37]. A comparative analysis of ABC/2 and volumetric methods identified 28–45 mL as the threshold for considering surgical intervention due to mass effect [25]. Based on ROC analysis, approximately 31 cm^3^ has also been proposed as a cutoff for SDC candidacy [23]. Collectively, although thresholds vary, most studies converge on a range of 25–35 cm^3^ as a reasonable criterion for surgical consideration.

In summary, the management of obstructive hydrocephalus secondary to cerebellar infarction can be guided by a pragmatic stepwise approach:

Initiate cautious CSF drainage with EVD and evaluate for clinical improvement.

If no clinical improvement is observed despite EVD, and infarct volume exceeds approximately 35 mL, proceed to SDC.

Consider infarct volume, lesion location, concomitant brainstem infarction, and overall clinical status together in surgical decision-making.

EVD should no longer be considered an absolute contraindication; rather, it serves as a bridging therapy in the modern management paradigm. This reflects a paradigm shift in neurosurgical practice, where EVD is now recognized as a controlled and safe option when properly titrated. Such reconsideration has meaningful implications for treatment algorithms and guideline interpretation. Future studies are needed to standardize drainage strategies and define surgical indications more precisely.

This scoping review has several limitations. First, by design, it favors breadth over depth and does not evaluate effect sizes or statistical significance, as no quantitative pooling or meta-analysis was performed. This approach is consistent with the PRISMA-ScR framework, which aims to comprehensively map the available evidence rather than to generate statistical estimates of treatment effects. Accordingly, our findings should be interpreted as descriptive rather than inferential. Second, the overall quality of the available evidence is limited. Most of the included studies were retrospective or observational in nature, frequently involving small sample sizes, heterogeneous inclusion criteria, and variable definitions of radiological or clinical endpoints. The absence of randomized controlled trials (RCTs) inherently introduces selection bias, as the choice between external ventricular drainage (EVD) and suboccipital decompressive craniectomy (SDC) is strongly influenced by initial neurological status, infarct characteristics, and institutional protocols. In clinical reality, more severe cases are typically selected for decompression, while EVD tends to be used in relatively stable patients. This bias may have contributed to apparent outcome differences observed in the literature. Third, potential publication bias must also be considered. Reports of favorable or successful EVD cases are more likely to be published, whereas complex or unfavorable cases may remain unpublished. Such bias may overestimate the perceived safety and efficacy of EVD in cerebellar infarction with obstructive hydrocephalus. Fourth, this review did not include a formal risk of bias or quality appraisal for the included studies. Although this omission aligns with the methodological intent of scoping reviews—which focus on mapping evidence rather than critically appraising it—it nevertheless limits the interpretability and generalizability of the findings. We acknowledge that incorporating a structured quality assessment in future systematic reviews or meta-analyses would help contextualize results derived from such diverse data sources. Fifth, the inclusion of a wide range of study designs—from multicenter registries and cohort analyses to small case series and isolated case reports—without explicit weighting may obscure the relative contribution of each evidence type. While this inclusivity broadens the overview, it also dilutes the strength of conclusions drawn from higher-quality studies. Finally, although we propose a practical stepwise management concept—beginning with cautious EVD and escalating to SDC when indicated—it should be regarded as a pragmatic synthesis derived from currently available, low-level evidence. This conceptual framework reflects accumulated clinical experience rather than a universally validated protocol. Its applicability may vary depending on local neurosurgical expertise, monitoring capabilities, and institutional resources. Therefore, we emphasize that our stepwise approach serves as a guide to facilitate individualized decision-making rather than as a prescriptive treatment algorithm. Despite these limitations, this review provides a structured synthesis of four decades of clinical data and evolving guideline perspectives, offering a consolidated foundation for future prospective multicenter investigations. Rigorous, standardized studies are needed to define volumetric thresholds, optimize drainage strategies, and clarify surgical timing in the management of cerebellar infarction with obstructive hydrocephalus.

## 5. Conclusions

Cerebellar infarction with obstructive hydrocephalus is a life-threatening emergency that requires prompt recognition and appropriate surgical management. Based on available evidence, the risk of upward transtentorial herniation may be lower than previously assumed; however, this interpretation is limited by the heterogeneity and low quality of existing studies and by the absence of standardized radiographic criteria. EVD may be used in selected patients with careful control of drainage parameters, while SDC remains essential in those with severe mass effect or brainstem compression. The diagnostic distinction of upward herniation—radiographically and clinically—remains challenging and should temper conclusions about EVD safety. The main contribution of this scoping review is to delineate current evidence, identify knowledge gaps, and determine whether a formal systematic review or prospective multicenter study is warranted to refine surgical indications and drainage strategies.

## Data Availability

The data used in this study are available from the corresponding author upon request.

## Supplementary Materials

The following supporting information can be downloaded at: https://www.mdpi.com/article/10.3390/jcm14248663/s1, Table S1. Summary of included studies on EVD with/without SDC for cerebellar infarction with obstructive hydrocephalus.
