# Supplementary material for: External Ventricular Drainage for Hydrocephalus Following Cerebellar Infarction: A Scoping Review
Source: J Clin Med. 2025 Dec 6;14(24):8663. doi: 10.3390/jcm14248663 (PMC12733857; doi:10.3390/jcm14248663)
Supplement: Supplementary file 1 [file jcm-14-08663-s001.zip › jcm-3943336-supplementary.pdf]

Supplementary Table S1. Summary of Included Studies on EVD with/without SDC for Cerebellar Infarction with Obstructive Hydrocephalus.

Author/Year	Study Design	Patient Characteristics	Pathophysiological Summary	Imaging Findings	Intervention	Outcome	Upward Herniation	Authors' Conclusion
**Lehrich JR et al., 1970 [24]**	Case series (4 cases) + literature review (16 cases analyzed)	Four institutional cases (1960–1968) + 12 from the literature. All had cerebellar hemispheric infarction acting as a posterior fossa mass lesion causing brainstem compression.	Typical course: progressive intracranial hypertension → decreased consciousness → coma, with ocular movement disorders and bilateral Babinski signs preceding decerebrate rigidity.	Reports of elevated intracranial pressure and lateral ventricular dilatation; fourth ventricle or aqueductal obstruction confirmed by pneumo- or ventriculography.	Emergency suboccipital craniectomy and partial resection of infarcted tissue; in some cases, high ventricular pressure confirmed by puncture before surgery. Rapid decompression recommended.	Among 13 surgically treated patients, 9 survived to discharge; most untreated patients progressed to coma and death.	Tonsillar herniation confirmed in 3 autopsies and 6 intraoperative cases. Upward herniation after EVD not described.	Emphasized that cerebellar infarction may behave like a posterior fossa tumor requiring urgent surgical decompression for survival.
**Woodhurst WB, 1980 [25]**	Single-center retrospective series	Six adult patients (4 men, 2 women) treated at Vancouver General Hospital (1977–1979).	Acute onset of vertigo, ataxia, headache, and vomiting progressing to brainstem compression (ocular motor palsy, coma, quadriplegia).	All patients underwent CT. Early CT may appear normal; low-density lesions became evident by day 5. Five of six developed hydrocephalus.	Two patients managed conservatively recovered; four underwent suboccipital craniectomy and infarct removal; CSF drainage used in two cases (EVD or lumbar drainage).	Two conservatively treated patients fully recovered; two surgically treated had mild deficits; two died (coma, postoperative infection).	No explicit mention of "upward herniation"; deterioration attributed mainly to hydrocephalus and brainstem compression.	Even initially mild cerebellar infarction can rapidly progress to a space-occupying lesion; early recognition and decompression are crucial.
**Taneda M et al., 1982 [26]**	Single-center retrospective observational series	15 cases with obstructive hydrocephalus among 1700 acute cerebrovascular patients (13 men, 2 women, aged 40–70s).	Large cerebellar infarction due to vertebrobasilar occlusion; edema and hemorrhagic transformation causing fourth ventricle obstruction and acute hydrocephalus.	CT showed hypo-, iso-, or hyperdense lesions depending on hemorrhagic changes. Hydrocephalus and fourth ventricle obstruction confirmed. Angiography demonstrated vertebrobasilar occlusion.	Ten patients underwent suboccipital decompression (3 after EVD); five managed conservatively.	Surgical group: 9/10 survived (6 full recovery, 1 partial, 2 severe disability); all conservatively treated patients died.	Brainstem infarction cases had poor prognosis even after hydrocephalus relief; EVD alone was insufficient for extensive infarction.	Massive cerebellar infarction is rare but fatal; prompt diagnosis and decompression can be lifesaving. EVD may aid diagnosis or bridge to surgery.
**Shenkin HA et al., 1982 [27]**	Retrospective single-center CT-based series	70 cases (15 cerebellar hemorrhage, 55 infarction) diagnosed by CT between 1977–1981.	Hydrocephalus strongly influenced prognosis; when present, consciousness deteriorated rapidly due to brainstem compression.	CT showed early hydrocephalus (day 0–2) in hemorrhage cases, and delayed onset (2–7 days) in infarctions.	12 cases with hydrocephalus (6 hemorrhage, 6 infarction) underwent EVD via Rickham reservoir.	Of 6 hemorrhage cases with EVD: 4 survived (2 with severe deficits), 2 died (decerebrate posture, fixed pupils). Of 6 infarction cases with EVD: 2 survived (1 mild deficit), 4 died.	The paper mentioned "upward herniation" only in reference to prior literature, denying its occurrence in their series.	Surgery unnecessary without hydrocephalus; when hydrocephalus develops, surgical drainage is mandatory for survival.
**Cioffi FA et al., 1985 [28]**	Prospective institutional series	Ten patients (mean age 54, range 38–80;	Acute cerebellar infarction presenting with	CT revealed low-density le-	All received medical management and	At discharge: 6 full recovery, 3 partial deficits,	One case suspected of transtentorial	Treatment algorithm: (1) Stable

		male predominance 3:2).	headache, vomiting, vertigo, and ataxia; some progressed to decreased consciousness.	sions with enhancement, fourth ventricle compression, hydrocephalus (4 cases), and mass effect (6 cases).	ICP monitoring; 5 underwent EVD, 3 later required CSF shunting, 1 underwent suboccipital craniectomy.	1 death (acute respiratory failure after EVD).	upward herniation after EVD.	with good consciousness – conservative ± ICP monitoring; (2) Stable but with hydrocephalus – conservative + EVD; (3) Deteriorating – EVD and/or decompression as indicated.
**Auer LM et al., 1986 [29]**	Retrospective single-center observational study	56 patients (40 infarction, 16 hemorrhage), 1977–1984, post-CT era consecutive cases	13/40 cerebellar infarction patients and 12/16 hemorrhage patients deteriorated due to brainstem compression and acute hydrocephalus.	CT: cerebellar hematomas (15–50 mL) with 75% ventricular rupture; infarctions showed unilateral low density with mass effect.	Hemorrhage: 10 early decompressions with EVD (2 EVD only), 4 conservative. Infarction: 7 suboccipital decompressions, others conservative.	Infarction: All 7 patients in the suboccipital decompression group survived. Patients with EVD alone died. 4 out of 5 patients in the conservative group died.	Warned that EVD alone may precipitate upward herniation in large lesions.	Both infarction and hemorrhage with consciousness decline should undergo prompt decompression; EVD serves for temporary ICP relief.
**Macdonell RA et al., 1987 [30]**	Retrospective study with autopsy correlation	30 cerebellar infarctions among 2000 stroke unit cases; mean age 66 years.	Symptoms: vertigo (80%), ataxia (77%), vomiting (63%), dysarthria (60%), headache (40%). Hydrocephalus in 13%.	CT positive in 6/11 within 24 h; diagnostic yield increased after 2–7 days.	Of 4 with hydrocephalus, 3 underwent suboccipital decompression (2 survived, 1 died).	In-hospital mortality 23%; deaths mainly from brainstem infarction.	Explicitly warned that EVD alone may trigger upward herniation; not recommended.	Cerebellar infarction has higher mortality; brainstem infarction determines prognosis. Early CT detection of hydrocephalus is vital.
**Bertalanffy H et al., 1992 [31]**	Retrospective case series	10 patients (1984–1989, age 42–77) with acute cerebellar infarction and obstructive hydrocephalus.	All cases: fourth ventricle/aqueduct obstruction → acute occlusive hydrocephalus.	CT: large cerebellar infarction, quadrigeminal cistern and ambient cistern obstruction, brainstem compression, ventricular enlargement.	6 patients had temporary ventriculostomy; 4 required permanent shunt (V-A or V-P).	9/10 improved (consciousness recovery or stabilization); 1 died due to multiple infarctions.	The authors argued that ventriculostomy alone, under local anesthesia, can be lifesaving without need for decompression.	CSF drainage alone was effective in most cases; suboccipital decompression rarely necessary.
**Kase CS et al., 1993 [15]**	Multicenter retrospective analysis (5 institutions)	66 cases (PICA: 36, SCA: 30), age 18–88 (mean 61), 46 men, 20 women.	PICA infarction caused severe edema and hydrocephalus, SCA infarction milder.	CT/MRI confirmed diagnosis; 25% of PICA infarctions involved total territory with marked mass effect.	Among 7 PICA infarctions with hydrocephalus: 3 died untreated; 4 had surgical drainage or decompression.	Mortality: PICA 17%, SCA 7%. Half of survivors had residual deficits.	No explicit mention of upward herniation; deterioration mainly due to brainstem compression.	PICA infarction tends to be severe and fatal; early detection and decompression improve survival.
**Rieke K et al., 1993 [9]**	Mixed retrospective (27) and prospective (15) single-center cohort (Heidelberg)	42 space-occupying cerebellar infarctions.	Mass effect and hydrocephalus with brainstem compression; BAEP/SSEP used for severity grading.	CT/MRI: fourth ventricle compression, hydrocephalus, and brainstem shift.	Group 1: conservative; Group 2: hydrocephalus → EVD; Group 3: coma with brainstem signs → SDC.	Good outcome (GOS 4–5): conservative 11/13, EVD 5/11, SDC 9/18.	No direct report of upward herniation, but EVD alone may be fatal in comatose brainstem-compressed patients.	Mild cases respond to conservative therapy; EVD effective when brainstem compression absent; SDC required in coma.

**Hornig CR et al., 1994 [4]**	Multicenter retrospective study	52 patients, mean age 61 (range 38–82), 65% male.	Space-occupying cerebellar infarction with fourth ventricle obstruction, hydrocephalus, and basal cistern compression.	CT: displacement of fourth ventricle, ventricular dilatation, basal cistern effacement (hydrocephalus in 81%).	16 conservative, 30 decompressive (22 with EVD, 12 with tonsillectomy), 10 excluded.	Outcome: 29 good recovery, 15 disabled, 8 dead. Even comatose cases improved with SDC.	"Upward herniation" not explicitly described; some EVD-only patients deteriorated before decompression.	SDC is first-line for progressive brainstem signs; EVD alone insufficient for decompression.
**Turgut M et al., 1996 [32]**	Retrospective single-center study	60 cases (39 hemorrhage, 21 infarction), 1976–1993.	Hypertension most common cause; clinical differentiation between hemorrhage and infarction difficult.	CT-based classification: <3 cm, 3–5 cm, >5 cm lesions. MRI used in 6 recent cases.	Hemorrhage: 22 decompressive, 17 conservative; Infarction: mostly conservative, 2 decompressive (misdiagnosed as tumor).	Hemorrhage mortality: 1/22 (surgery), 6/17 (conservative). Infarction mortality: 1/21 (>5 cm).	No mention of upward herniation; deterioration linked to lesion size and brainstem compression.	Small lesions favorable conservatively; large lesions require early surgery for survival.
**Jauss M et al., 1999 [1]**	Prospective multicenter observational study (17 centers, 1992–1996)	84 patients (age 22–78, mean 58.5) with large cerebellar infarction showing mass effect on CT.	Neurological worsening occurred on day 2–4 (median 3). Predictors: infarct volume, mass effect, and hydrocephalus.	CT scoring (0–9): fourth ventricle compression, quadrigeminal cistern closure, temporal horn dilatation.	34 SDC, 14 EVD, 36 conservative. Four EVD cases later required SDC.	Good outcome (mRS ≤2 at 3 months): medical 83%, EVD 71%, SDC 65%.	No quantitative data on upward herniation, though ventricular drainage recognized as risk if excessive.	Surgical intervention is life-saving in comatose cases; conservative management suitable for stable patients.
**Jauss M et al., 2001 [14]**	Prospective analysis (German–Austrian Cerebellar Infarction Study)	84 large cerebellar infarctions; mean age 59; etiologies: cardioembolic 25, dissection 1, undetermined 58.	Mass effect and hydrocephalus were major predictors of deterioration; CT evaluation crucial in obtunded patients.	CT score (0–9) with excellent interobserver reliability.	36 conservative, 14 EVD, 34 SDC. Postoperative CT scores improved significantly (5→3 →2).	CT scores correlated strongly with consciousness level; postoperative improvement reflected decompression efficacy.	No quantitative herniation data, but progression with hydrocephalus recognized as risk factor.	CT scoring provides objective monitoring of mass effect and treatment response in cerebellar infarction.
**Raco A et al., 2003 [8]**	Retrospective cohort (8 years, 1991–1998)	44 cases (24 men, 20 women; mean 56 years; age 9–83), excluding brainstem invasion.	Cerebellar infarction with deterioration and hydrocephalus; symptoms included vertigo, nausea/vomiting, headache, ataxia.	CT/MRI: fourth ventricle deviation ≥3 mm, brainstem deformation, tight posterior fossa.	25 conservative, 8 EVD only, 5 EVD→SDC, 4 SDC only, 2 treatment-limited.	Outcomes: conservative 20 good/4 moderate/1 death; EVD only 7 good/1 moderate; EVD+SDC 3 good/1 moderate; SDC 3 good/1 death.	Authors stated that EVD-related herniation risk can be minimized with drainage control; no clinical cases observed.	Stepwise strategy: EVD first for conscious hydrocephalus; proceed to SDC if no improvement.
**Baldauf J et al., 2006 [33]**	Retrospective single-center series	10 patients (age 25–85, mean 62; M:F = 6:4); mean 4 days from onset to surgery.	Obstructive hydrocephalus secondary to cerebellar infarction (PICA: 6, SCA: 2, multiterritory: 2).	CT: fourth ventricle obstruction, ventricular dilation, moderate-to-severe mass effect in all cases.	9 underwent endoscopic third ventriculostomy (ETV) as primary treatment; 1 required EVD after failed ETV.	8 patients improved in consciousness; mean GOS at discharge 3.4; 4 regained functional independence.	Not specified; ETV selected to avoid EVD-related infection risk.	ETV is a safe and effective alternative for obstructive hydrocephalus secondary to cerebellar infarction, reducing infection risk compared with EVD.
**Kudo H et al., 2007 [16]**	Multicenter retrospective study (4 hospitals, 1994–2004)	25 patients with severe cerebellar infarction (GCS < 9).	All had impaired consciousness from severe cerebellar infarction with brainstem compression	CT: fourth ventricle displacement/obstruction and brainstem compression; no unified imaging cutoff.	Group A: 3 EVD only, 2 EVD → resection next day; Group B: 10 decompressions	Good outcomes in 10 decompression cases; poor in EVD-only. Mortality	No clinical upward herniation observed, though EVD alone considered inadequate.	In severe cerebellar infarction (GCS < 9), EVD alone leads to poor prognosis;

			and hydrocephalus.		(EVD ± lesionectomy).	higher in delayed interventions.		early decompression is recommended.
**Pfefferkorn T et al., 2009 [17]**	Multicenter retrospective cohort (Germany, 10 years)	57 cases of malignant cerebellar infarction (defined by mass effect and hydrocephalus).	Rapid neurological deterioration from mass effect and obstructive hydrocephalus.	MRI/CT: quantified fourth ventricle obstruction and brainstem compression.	Conservative, EVD only, or SDC; 10 had EVD only, SDC reserved for more severe cases.	12-month mRS 0–2 significantly higher in surgical group (SDC ± EVD); EVD-only associated with poor outcome.	No direct data, but EVD alone considered insufficient to relieve brainstem compression.	Surgical decompression is life-saving in malignant cerebellar infarction; EVD alone often inadequate.
**Jüttler E et al., 2009 [34]**	Retrospective single-center cohort (1996–2005)	56 patients with space-occupying cerebellar infarction.	Severe mass effect causing fourth ventricle obstruction, hydrocephalus, and brainstem compression.	Jauss CT score (0–9): median 6 (range 4–9).	9 EVD (16%), 8 SDC (14%), 39 combined SDC + EVD (70%).	Median follow-up 8.2 years: survival 60.7%; good outcome (mRS 0–2) in 35.7%.	Not directly analyzed; discussion notes risk of excessive CSF drainage.	Long-term prognosis after surgical treatment not uniformly favorable; mortality remains high despite aggressive intervention.
**Tsitsopoulos PP et al., 2011 [35]**	Retrospective single-center series (1999–2007)	10 patients with bilateral expansive cerebellar infarction; 6 had concurrent brainstem infarction.	Bilateral infarction causing severe mass effect, fourth ventricle obstruction, and hydrocephalus.	Jauss CT score mean 5.2 ± 2.2; hydrocephalus in 7 cases.	All underwent EVD + bilateral suboccipital decompression with infarct removal; 4 had C1 laminectomy.	Mean GCS improved from 8.9 to 12.6; mean mRS 2.8 at long-term follow-up (median 58 months).	Not quantified, but authors note EVD alone is insufficient to prevent herniation.	Combined EVD, wide decompression, and infarct removal can achieve favorable outcomes in bilateral infarction.
**Tchopev Z et al., 2013 [36]**	Retrospective single-center cohort (2007–2011)	44 cases of cerebellar infarction (mean age 55; 68% male).	Mass effect and hydrocephalus; brainstem infarction in 29.5%.	DWI and ADC used to quantify infarct volume and rVolume (infarct/cerebellum ratio).	Mainly conservative management; EVD/SDC performed in poor-outcome subgroup.	Poor outcome in 29.5%; infarct volume 28.4 mL (vs 11.7 mL in good outcome).	No direct herniation data; deterioration linked to mass effect.	DWI-derived infarct volume (25–30 mL, rVolume 25–30%) predicts poor outcome and surgical need.
**Mostofi K, 2013 [10]**	Retrospective single-center cohort (2000–2008)	53 patients with massive ischemic cerebellar infarction (MICI).	Large infarct (>5 cm³) with hydrocephalus and brainstem compression.	CT/MRI to measure infarct volume and assess hydrocephalus.	Surgery (SODC 16, EVD 6, SODC + EVD 3) vs conservative (n=28).	Surgical group: 8% mortality; conservative: 4 deaths among comatose cases.	No quantitative data; noted that EVD alone cannot relieve mass effect.	Early surgery, particularly SODC, improves survival in MICI; EVD alone insufficient.
**Neugebauer H et al., 2013 [2]**	Systematic literature review (PubMed/Medline up to 2012)	115 articles, >750 total cases of space-occupying cerebellar infarction.	Cerebellar swelling → brainstem compression, fourth ventricle obstruction → hydrocephalus; fatal when herniation occurs.	CT less sensitive; MRI superior. Jauss CT score referenced for mass effect.	Conservative management, EVD, SDC, or lesionectomy described across studies.	Conservative mortality 42.9%; EVD survival 81.6%; SDC 76.8%.	Upward herniation a classical concern but rarely fatal in modern EVD practice.	Surgical intervention (SDC ± EVD) is lifesaving, though prognosis depends on age and brainstem involvement.
**Agarwalla PK et al., 2014 [5]**	Narrative review (1908–2013, anterior vs posterior circulation decompression studies)	Compilation of published studies on decompressive craniectomy for cerebellar infarction.	Mass effect from infarction leads to brainstem compression and acute hydrocephalus.	Summarized CT parameters (fourth ventricle obstruction, cistern effacement, ventricular dilation).	Reviewed techniques: SDC ± EVD, ± infarct removal, C1 laminectomy.	SDC demonstrated life-saving potential; long-term outcomes better in younger, brainstem-intact patients.	Cited van Loon (1993): overdrainage during EVD can rarely cause upward herniation.	SDC remains effective; decision should consider consciousness level, imaging mass effect, and brainstem ischemia.

**Puffer RC et al., 2016 [37]**	Retrospective single-center cohort	34 patients ≥60 years (2000–2014): 29 hemorrhage, 5 infarction; all underwent emergency posterior fossa decompression.	Rapid deterioration due to cerebellar infarction or hemorrhage with hydrocephalus.	Preoperative CT/MRI evaluated lesion volume, brainstem compression, and hydrocephalus; 55% had EVD.	All underwent posterior fossa decompression ± EVD.	30-day mortality: 0% (60–69 y), 33% (70–79 y), 25% (≥80 y); 1-year mortality 47%.	Not specified; EVD use not associated with increased mortality.	In elderly patients, mortality remains high and postoperative independence limited; minimal decompression should be considered.
**Kim MJ et al., 2016 [18]**	Multicenter retrospective matched case-control study	From a database of 721 cerebellar infarctions, 28 underwent preventive SDC (Group A) vs matched controls (Group B).	Progressive mass effect, hydrocephalus, and brainstem compression within 72 h of onset.	MRI: infarct-to-cerebellar volume ratio 0.25–0.33; selected for preventive SDC.	Group A: preventive SDC (EVD in 50%); Group B: standard treatment.	12-month good outcome (mRS 0–2): 66.7% (SDC) vs 51.0% (control), *p* = 0.047.	Not directly reported; text warns that EVD alone may precipitate upward herniation.	Preventive SDC improved survival and function in patients without brainstem infarction and infarct ratio 0.25–0.33.
**Tartara F et al., 2018 [19]**	Multicenter retrospective case series (7 centers, Italy)	11 patients (mean age 64.7); 9 PICA, 2 multiterritory infarctions.	Mass effect and acute obstructive hydrocephalus; EVD performed in 82%.	CT: fourth ventricle obstruction, brainstem compression, tonsillar descent.	Small suboccipital craniectomy with strokectomy and cisterna magna drainage.	6-month mRS ≤ 2 in 82%; one death (myocardial infarction).	Not specified; discussion notes risk of upward herniation with isolated EVD.	Limited craniectomy with strokectomy effectively relieves both compression and hydrocephalus.
**Braksick SA et al., 2018 [11]**	Retrospective single-center cohort (2007–2014, Mayo Clinic)	25 posterior fossa lesions with obstructive hydrocephalus (2 cerebellar infarctions).	Acute obstructive hydrocephalus; many were comatose before EVD.	CT/MRI: upward herniation in 88% pre-EVD (22/25); 2 worsened post-EVD.	All underwent emergency EVD; GCS changes assessed pre/post.	GCS improved or stabilized in 92% (48% improved, 44% unchanged).	Imaging-confirmed upward herniation pre-EVD in 88%; post-EVD worsening rare (2/25).	Upward herniation often precedes EVD; post-EVD deterioration uncommon with careful management.
**Lindeskog D et al., 2019 [38]**	Retrospective single-center cohort	22 SDC-treated cerebellar infarctions (median age 53).	Space-occupying infarction with hydrocephalus and brainstem compression; 9 received prior EVD.	CT/MRI: PICA (19), SCA (10), AICA (8); bilateral infarction 6; brainstem involvement noted.	All underwent SDC (9 with prior EVD); 14 required infarct evacuation; 2 had C1 laminectomy.	At 1 year, median mRS 3; good outcome (mRS 0–3) 54%.	Not reported.	SDC is lifesaving; half of patients achieve good long-term recovery; poor outcomes linked to bilateral or brainstem infarction.
**Hernández-Durán S et al., 2020 [39]**	Retrospective single-center cohort (2010–2018)	34 patients (median age 70; 74% unilateral, 26% bilateral).	Space-occupying cerebellar infarction treated by necrosectomy; indication: lesion >3 cm or clinical decline.	CT: infarct volume measurement; fourth ventricle closure, basal cistern effacement, acute hydrocephalus (56%).	Suboccipital craniectomy + necrosectomy; 12 with EVD (4 pre-, 8 post-op).	Mortality 21%; good outcome (GOS ≥ 4, mRS ≤ 2, BI ≥ 90) in 76%.	Not specified; EVD avoided as sole therapy.	Necrosectomy is a viable alternative to SDC, with comparable survival and functional outcomes.
**Taylor DR et al., 2020 [40]**	Retrospective single-center cohort (2011–2016, USA, n=86)	Median age 58.5; 50% PICA infarction; comorbidities: HT 73%, DM 34%.	Hydrocephalus in 18.6%, brainstem compression 20.9%, infarction 22.1%.	CT/MRI: CIS score (ventricle/cistern involvement at 5 sites: 4th ventricle, prepontine cistern, quadrigeminal cistern, tonsillar level, lateral ventricle).	21 surgical cases (24.4%): 18 EVD, 9 SDC+EVD, 3 SDC only, 5 preventive.	Mortality 23%; intervention group had larger/faster edema; longer stay but improved survival.	Not reported.	CIS score correlates with need for surgery and edema progression; may guide timely intervention.

**Broocks G et al., 2021 [20]**	Multicenter retrospective cohort (2010–2019)	179 posterior circulation strokes, 35 with malignant cerebellar edema (MCE).	MCE defined by mass effect and hydrocephalus; predictive markers unclear.	pcNWU (posterior circulation net water uptake) measured on early CT.	Conservative or endovascular therapy; 8 underwent SDC.	Mortality: MCE 60% vs non-MCE 17%; median mRS 6 vs 3.5.	Not reported.	pcNWU on initial CT strongly predicts MCE (AUC 0.94); may aid early triage for decompression.
**Lim NA et al., 2023 [43]**	Systematic review (MEDLINE, EMBASE, Cochrane, to Apr 2021; 31 studies)	723 posterior fossa infarctions (184 surgical vs 235 conservative, +2 case series).	Malignant posterior circulation infarction (MPCI) with mass effect and hydrocephalus.	Radiologic criteria: 4th ventricle compression, hydrocephalus, brainstem shift, basal cistern effacement.	Surgery: EVD, SDC, SDC + necrosectomy, ETV, or shunt; vs conservative management.	Surgery reduced mortality (e.g., 66% → ~20%) and improved GCS; functional outcome benefit uncertain.	Not reported.	Surgery improves survival in severe MPCI, though evidence for functional benefit remains limited.
**Krishnan K et al., 2023 [44]**	Narrative review	Compilation of observational and meta-analytic studies on decompression for space-occupying cerebellar infarction.	SOCS causes brainstem compression and obstructive hydrocephalus, often fatal without intervention.	CT/MRI indicators: 4th ventricle closure, brainstem compression, mass effect.	Discussed DSC ± EVD; isolated EVD carries theoretical risk.	Meta-analysis (n=283): mortality 19%, moderate–severe disability 28%; surgery within 48 h best.	Theoretical risk of upward herniation with isolated EVD.	Decompressive surgery (±EVD) is lifesaving, though functional recovery evidence remains limited.
**Won SY et al., 2023 [21]**	Multicenter retrospective cohort (2008–2021, 5 German centers)	531 ischemic cerebellar infarctions (mean age 68.7; 56.7% female); 127 surgical (23.9%).	Evaluated 30-day mortality and poor outcome predictors; developed CS and CS-GS scores.	MRI: infarct volume median 17.1 cm^3 (0.1–88.8), brainstem involvement presence/absence.	Conservative vs surgical (SDC ± EVD); EVD-only classified as conservative.	30-day mortality 9.8%; poor outcome (mRS 4–6) 28.1%; predictors: age ≥70, low admission GCS, bilateral infarction.	Not reported.	Newly proposed CS and CS-GS scores accurately predict 30-day mortality and poor outcomes.
**Won SY et al., 2024 [22]**	Multicenter retrospective cohort (Germany, 5 centers; 2008–2021; n=531)	Mean age 68; 24% surgical (SDC ± EVD) vs 76% conservative; GCS and infarct volume recorded.	Space-occupying cerebellar infarction (SOCS) with hydrocephalus/brainstem compression; treatment-effect heterogeneity by infarct size.	MRI-based infarct volume; brainstem involvement documented.	Conservative vs SDC ± EVD (EVD-only counted as conservative).	Overall, no crude outcome difference; volume-dependent crossover effect: ≥35 mL favored surgery, <35 mL favored conservative care.	Not reported.	Surgical benefit depends on infarct volume; consider ≥35 mL as a pragmatic threshold for offering decompression.
**Hernandez-Duran S et al., 2024 [23]**	Multicenter retrospective cohort (2011–2021; n=91)	SOCS treated surgically; necrosectomy vs SDC.	Decompression plus >50% necrotic tissue removal linked to improved recovery.	Post-op residual volume quantified; threshold ≤17 cm^3 associated with better outcomes.	SDC with or without necrosectomy; goal to minimize residual mass effect.	Lower in-hospital mortality and better discharge status when post-op volume ≤17 cm^3 or >50% necrosis removed.	Not reported.	Aim for postoperative volume ≤17 cm^3 or >50% necrosectomy to optimize outcomes in SOCS.
**Nesa AS et al., 2024 [41]**	Single-center retrospective cohort (2014–2020)	19 severe SOCS patients undergoing SDC + EVD; high comorbidity burden.	Malignant edema with obstructive hydrocephalus requiring combined decompression and CSF diversion.	Standard CT/MRI criteria for mass effect/hydrocephalus.	Uniform SDC + EVD strategy; limited use of medical-only therapy.	Poor 6-month functional outcomes (death or severe disability in all cases).	Not reported.	In a very severe cohort, SDC + EVD did not translate into good functional recovery; careful selection remains crucial.
**Arsenovic M et al., 2025 [42]**	Retrospective single-center analysis	Consecutive SOCS cases; compared volumetry by ABC/2 vs software.	Practical volume estimation needed to guide surgical thresholds.	ABC/2 closely matched software volumes (e.g., 16.6 vs 15.9 mL; ns).	Imaging-driven triage; not an interventional study.	Not an outcome study.	Not reported.	ABC/2 is clinically reliable for posterior fossa infarct volumetry and suitable for threshold-

								based decision-making (e.g., ≥35 mL).
**Hernandez-Duran S et al., 2025 [45]**	International cross-sectional online survey (Oct–Dec 2024)	Neurosurgeons/intensivists worldwide; practices for SOCS management.	Wide variation in indications, timing, and techniques; limited standardization.	—	Reported use of SDC, necrosectomy, EVD, and shunting; EVD rarely used as sole definitive therapy.	No clinical outcomes collected.	Noted skepticism toward EVD-only; typically used as an adjunct/bridge.	Global practice is heterogeneous; standardized criteria (volume, consciousness, brainstem signs) are needed to harmonize care.

Abbreviations: CT—computed tomography; MRI—magnetic resonance imaging; EVD—external ventricular drainage; SDC—suboccipital decompressive craniectomy; GCS—Glasgow Coma Scale; mRS—modified Rankin Scale; GOS—Glasgow Outcome Scale; ICP—intracranial pressure; PICA—posterior inferior cerebellar artery; SCA—superior cerebellar artery; AICA—anterior inferior cerebellar artery; BAEP—brainstem auditory evoked potential; DWI—diffusion-weighted imaging; ADC—apparent diffusion coefficient; ETV—endoscopic third ventriculostomy; ROC—receiver operating characteristic; BI—Barthel Index; NIHSS—National Institutes of Health Stroke Scale; CSF—cerebrospinal fluid; SOCS—space-occupying cerebellar stroke; MCE—malignant cerebellar edema; CIS—cisternal involvement score; pcNWU—posterior circulation net water uptake; V-P—ventriculoperitoneal shunt; V-A—ventriculoatrial shunt.

 https://doi.org/10.3390/jcm14248663
